# Cotton textiles modified with citric acid as efficient anti-bacterial agent for prevention of nosocomial infections

**DOI:** 10.3325/cmj.2011.52.68

**Published:** 2011-02

**Authors:** Sandra Bischof Vukušić, Sandra Flinčec Grgac, Ana Budimir, Smilja Kalenić

**Affiliations:** 1 University of Zagreb, Faculty of Textile Technology, Zagreb, Croatia; 2 Clinical Hospital Center, University of Zagreb, School of Medicine, Zagreb, Croatia

## Abstract

**Aim:**

To study the antimicrobial activity of citric acid (CA) and sodium hypophosphite monohydrate (SHP) against gram-positive and gram-negative bacteria*,* and to determine the influence of conventional and microwave thermal treatments on the effectiveness of antimicrobial treatment of cotton textiles.

**Method:**

Textile material was impregnated with CA and SHP solution and thermally treated by either conventional or microwave drying/curing treatment. Antibacterial effectiveness was tested according to the ISO 20743:2009 standard, using absorption method. The surfaces were morphologically observed by scanning electron microscopy, while physical characteristics were determined by wrinkle recovery angles method (DIN 53 891), tensile strength (DIN 53 837), and whiteness degree method (AATCC 110-2000).

**Results:**

Cotton fabric treated with CA and SHP showed significant antibacterial activity against MRSA (6.38 log_10_ treated by conventional drying and 6.46 log_10_ treated by microwave drying before washing, and 6.90 log_10_ and 7.86 log_10_, respectively, after 1 cycle of home domestic laundering washing [HDLW]). Antibacterial activity was also remarkable against *S. aureus* (4.25 log_10_ by conventional drying, 4.58 log_10_ by microwave drying) and against *P. aeruginosa* (1.93 log_10_ by conventional and 4.66 log_10_ by microwave drying). Antibacterial activity against *P. aeruginosa* was higher in samples subjected to microwave drying/curing than in those subjected to conventional drying/curing. As expected, antibacterial activity was reduced after 10 HDLW cycles but the compound was still effective. The surface of the untreated cotton polymer was smooth, while minor erosion stripes appeared on the surfaces treated with antimicrobial agent, and long and deep stripes were found on the surface of the washed sample.

**Conclusion:**

CA can be used both for the disposable (non-durable) materials (gowns, masks, and cuffs for blood pressure measurement) and the materials that require durability to laundering. The current protocols and initiatives in infection control could be improved by the use of antimicrobial agents applied on cotton carbohydrate polymer.

A leading cause of community-acquired infections and the first cause of nosocomially acquired bacteremia is *Staphylococcus aureus* (1). During the antibiotic era, strains of *S. aureus* have developed resistance to antimicrobial agents, such as methicillin and other beta-lactam antibiotics. Methicilin-resistant *Staphylococcus aureus* (MRSA) is a microorganism with very challenging treatment, eradication, and control. Since the discovery of the first clinical isolate almost 5 decades ago, MRSA strains have spread rapidly throughout the world (2), probably due to evolutionary changes in the microorganism in association with ineffective infection control practices and the intensified selective pressure fomented by increased antimicrobial use. The worldwide prevalence of MRSA in blood cultures varies from 0.6% in the Netherlands to 66.8% in Japan (3). It is also now clear that MRSA is not exclusively a hospital pathogen and that we have strains emerging in the community, causing serious infections. The organism’s resistance to methicillin is probably caused by a chromosomal gene, *mecA* (4,5). MRSA colonization and infection contributes to the increased overall cost of health care and its incidence has raised despite implementation of infection control programs. It is generally accepted that people serve as its major reservoir and that other sources for the microorganisms are relatively unimportant except in special circumstances (6,7). MRSA has been recovered from many surfaces in hospitals, including stethoscopes, floors, linens, air vents, charts, tourniquets, furniture, hydrotherapy tubs, bed sheets, and dry mops, but few reports have indicated that these are important in transmission. Environmental surfaces in burn units may be heavily contaminated by staphylococci and in this setting may constitute a very important reservoir (8-10).

*Pseudomonas aeruginosa* is a representative of typical nosocomial gram-negative pathogen with minimal growth requirements and potential for resistance development. It belongs to the family of *Pseudomonadaceae*, which are effective opportunistic pathogens and is primarily a nosocomial pathogen. Therefore, the frequency with which it causes disease can be reliably estimated from annual surveillance data collected by the Centers for Disease Control and Prevention National Nosocomial Infections Surveillance system. According to these data, collected between 1990 and 1996, *P. aeruginosa* was the second most common cause of nosocomial pneumonia (17% of isolates), the third most common cause of urinary tract infection (11%), the fourth most common cause of surgical site infection (8%), the seventh most frequently isolated pathogen from the blood stream (3%), and the fifth most common isolate (9%) overall, obtained from all sites (1). Human *Pseudomonas* disease is also associated with water-related reservoirs outside of hospitals, including swimming pools, whirlpool tubs, hot tubs, and contact lens solutions. Human colonization occurs at moist sites such as the perineum, axilla, and ear. Moisture is also a critical factor in hospital reservoirs of *P. aeruginosa*, such as respiratory equipment, clean solutions, medicines, disinfectants, sinks, mops, food mixers, and vegetables.

The primary, time-honored, principle of routine infection prevention and control is hand washing, which is considered effective for eliminating transient hand contamination with pathogens acquired from patients or the environment, such as MRSA (11). Nowadays, other numerous and cost-effective techniques have been recommended to eliminate the MRSA strain from hospitals, schools, and prisons, such as negative ion, UV & HEPA air purification systems, and cleaning with hydrogen peroxide or a silver solution.

In order to resolve health concerns or to reduce microbiologically-caused odor, deterioration, or staining of textile materials, antimicrobial finishing is used quite often today. Citric acid (CA) is already on the Environmental Protection Agency list of registered agents against MRSA, in combination with phosphoric acid. In this study, a different catalyst was applied – sodium hypophosphite monohydrate (SHP). CA is safe in process and use and can be applied easily and economically on textile fabrics. It is considered to be environmentally benign and its world-wide annual production stands at approximately 1 700 000 MT/y. More than 50% of its use amounts to beverages, some 20% to other food applications, 20% for detergent applications, and 10% for other non-food related applications like cosmetics and in the chemical industry. CA is organic acid and one of the polycarboxylic acids, which have been previously used as environmentally friendly Durable Press finishing agent (12-14).

Based on the mode of attack on microbes, CA can be considered to chemically bind to fiber surfaces and as such is a non-leaching type. Antimicrobial activity of organic acid is attributed to pH reduction, depression of internal pH of microbial cell by ionization of undissociated acid molecules, and disruption of substrate transport by altering cell membrane permeability (15) or reduction of proton motive force. The salts made in this reaction get in touch with the negatively charged protoplasm of the microorganisms and demolish the cell membrane. This collective procedure imparts finish that can tolerate 20 wash cycles along with tumble-drying (16). CA forms ester bonding with the cellulose hydroxyls through the formation of anhydrides (14).

An alternative approach investigated in this article is the application of microwave energy to impart improved fixation of antibacterial compounds, which will consequently lead to improved laundering durability of treated material. One of the most outstanding advantages of microwave technology is its volumetrical heating, minimizing the damage from over-drying. Since heat energy is transferred through the material electromagnetically, and not as a heat flux, it is not limited with the volume of the material. Microwave heating of textile fabrics is almost instantaneous throughout the material, so that the environment of the fabric insulates it against heat loss from the surface, allowing curing. In order to prevent hot spots which can cause burns, especially when light cloths are treated, fabrics must be kept in motion during the treatment to obtain effective curing (17).

The aim of this article was to study the antimicrobial activity of CA against two strains of gram-positive *S. aureus* bacterium and a gram-negative bacterium *P. aeruginosa*. The second aim was to determine the influence of the two different thermal treatments, conventional and microwave, on the effectiveness of antimicrobial treated cotton textiles.

## Methods

We used bleached cotton fabric in a plain weave, weighting 150 g/m^2^. To provide antimicrobial finish, the samples were impregnated with 7% CA and 6.5% SHP solution with ~ 100% wet pickup. The first part of the samples was dried at 110°C for 3 minutes and cured at 180°C for 90 seconds, while the second part was dried and cured by the microwaves of 900 W for 20 minutes. After the thermal treatments, samples were washed 1-10 times (home domestic laundering washing, HDLW) in accordance with EN ISO 69330 (2005) to test laundering durability.

**Figure 1:**
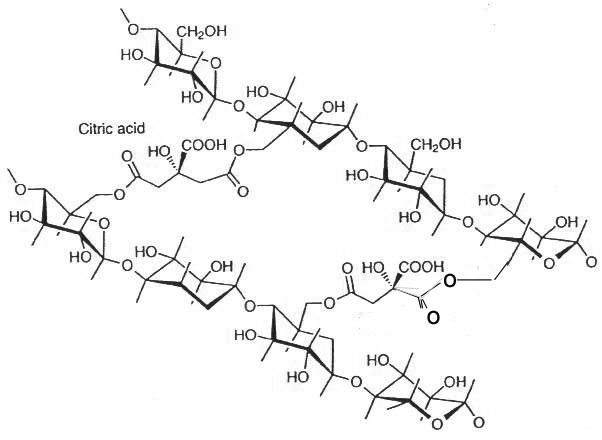
Microwave treatment scheme of the textile material passing through the waveguides; taken with permission from Katović et al, inventors (17).

After the impregnation, one part of the samples was thermally treated at the patented laboratory microwave device for treatment of textile materials (Figure 1). The microwave device consists of 6 rectangular waveguides centrally slotted in order to obtain planar passage of textile material in a wide state. In this way, the material lies in the maximum of the electric field, which assures effective coupling to the flowing microwave energy. Applied microwaves frequency was 2.45 GHz.

Determination of antibacterial activity was performed according to ISO 20743:2009 standard (18), specifically using absorption method, an evaluation method in which test bacterial suspension is inoculated directly on the samples. The first strain used in this method was COL-1 MRSA strain, which was isolated first in 1965 in the UK, known as a representative of Archaic clone (3). The strain has the following characteristics: presence of SCC*mec* (Staphylococcal Chromosomal Cassette mec) type I, ccrAB (Cassette Chromosome Recombinase AB) 1, and sequence type 4 (3). The second strain was *S. aureus* ATCC 6538, and the third strain was *P. aeruginosa* ATCC 27853.

Bacteria were grown on enumeration agar, pH = 7.2 ± 0.2, containing dehydrated yeast extract, casein tryptone, glucose, and agar. Afterwards, one colony was placed in 20 mL of nutrition broth, pH = 6.9 ± 0.2, composed of beef extract, peptone, and water, for 18-24 hours at 37°C. Inoculums from the previous step (0.4 mL) were placed in 20 mL of nutrition broth and incubated for additional 3 ± 1 hour in order to obtain the concentration of bacteria of 10^7^ colony forming units/mL. The prepared inoculums were preserved by ice-cooling and used within 8 hours for adjusting the bacteria to concentration of 1 × 10^5^ by McFarland’s nephelometer. A total of 0.2 mL of inoculums was pipetted at several points on each test sample. Immediately after inoculation, 20 mL of Soybean Casein Digest with Lecithin and Polysorbate formulated medium was added using the shake-out method (18), after the incubation for 24 hours at 37°C. A total of 1 mL of inoculums was taken from test sample and a serial of 10-fold dilutions were made. After vortexing, 1 mL from each dilution was added to 15 mL of warm (50°C) enumeration agar. After solidification, the plates were turned upside down and incubated at 37°C for 24 hours. Quantitative measurement was performed by plate count method. Judgment of test effectiveness was performed by the same ISO standard. Calculations of antibacterial activity value (G) included the difference between growth value of the control fabric (Tt) and growth value of antibacterial treated sample (To); G = Tt-To. The evaluation is done by counting the colony forming units and presented in logarithmic form (log stages).

Field emission scanning electron microscope (FE SEM, Mira II LMU, Tescan, Brno, Czech Republic) was used for sample analysis. The samples were coated with a conductive Ag/Pt layer and scanned under the conditions of high voltage (HV 10.00kV). Wrinkle recovery angles were determined according to DIN 53 891 and tensile strength according to DIN 53 837. Whiteness degree was measured with spectrophotometer (Spectraflash SF 300, Datacolor AG, Zurich, Switzerland) with Data Match 300 program according to AATCC 110-2000 standard.

## Results

The results of antibacterial effectiveness tested in triplicate for 3 bacteria are presented in Table 1. CA showed significant antibacterial activity against MRSA, *S. aureus* strain, and *P. aeruginosa*. Antibacterial activity against *P. aeruginosa* was higher in samples subjected to microwave drying/curing than in the samples subjected to conventional drying/curing (Table 1). As expected, antibacterial activity was reduced after 10 HDLW cycles but the compound was still effective.

**Table 1:** Antibacterial activity of tested cotton samples against three bacterial strains*^†^

		MRSA (Col-1)				*Staphylococcus aureus* (ATCC 6538)				*Pseudomonas aeruginosa* (ATCC 27853)			
		conventional drying		microwave drying		conventional drying		microwave drying		conventional drying		microwave drying	
Concentration of inoculums (CFU/mL)		1.8 × 10^5^		1.8 × 10^5^		1.8 × 10^5^		1.8 × 10^5^		1.7 × 10^5^		1.7 × 10^5^	
Difference of extremes for three control fabrics (lg)		0 h 0.02	24 h 1.74	0 h 0.04	24 h 1.52	0 h 0.98	24 h 0.53	0 h 0.47	24 h 1.07	0 h 0.04	24 h 1.12	0 h 2.23	24 h 0.21
Prior to washing	F = Ct-Co	2.06		2.06		0.87		0.86		1.67		2.56	
	G = Tt-To	-4.32		-4.40		-3.38		-3.72		-0.26		-2.10	
	A = F- G	6.38		6.46		4.25		4.58		1.93		4.66	
After 1 HDLW cycle	F = Ct- Co	2.06		2.06		0.92		0.98		1.67		2.56	
	G = Tt- To	-4.84		-5.80		-2.99		-3.34		-1.75		-2.09	
	A = F- G	6.90		7.86		3.91		4.32		3.42		4.65	
After 10 HDLW cycles	F = Ct- Co	1.08		1.08		0.87		1.58		2.31		2.31	
	G = Tt-To	-1.40		-1.63		-1.69		-1.83		2.87		2.67	
	A = F- G	2.48		2.71		2.56		3.41		-0.56		-0.36	

*Abbreviations: Ct – average common logarithm for the number of bacteria obtained from three test samples of control fabric after an 18-24 h incubation; Co – average common logarithm for the number of bacteria obtained from three test samples of control fabric immediately after inoculation; F – growth value on the control fabric; A – antibacterial activity value; G – growth value on the antibacterially treated sample; Tt – average number of bacteria obtained from three antibacterially treated test samples after an 18-24 h incubation; To – the number of bacteria obtained from three antibacterial-treated test samples immediately after transfer; MRSA – methicillin-resistant *Staphylococcus aureus*; HDLW – home domestic laundering washing; CFU – colony forming units.

†Sterilization method: autoclave. Incubation time 24 h.

CA showed significant antibacterial activity against MRSA, 6.38 log_10_ obtained by conventional drying and 6.46 log_10_ obtained by microwave drying prior to washing, and 6.90 log_10_ and 7.86 log_10_, respectively, after 1 HDLW. As expected, antibacterial activity was reduced after 10 HDLW cycles but the compound was still effective (Table 1). The samples dried either conventionally or by microwaves and tested after 10 HDLW cycles showed the growth value (G) of -1.4 and -1.63 and antibacterial activity value (A) of 2.48 and 2.71, respectively. This means that the difference in germ reduction between the treated/washed and untreated material was greater than 100 times (10^2^), which is sufficient for antibacterial purposes. Antimicrobial treatment of cotton samples showed excellent results against *S. aureus*, as well as against MRSA (Table 1). Reduction rate was lower only after 10 HDWL cycles, but still confirming excellent and durable antimicrobial protection. The results of microwave drying/curing treatment are even better (3.41) than that of conventional drying/curing treatment (2.56).

Antibacterial activity against *P. aeruginosa* in samples subjected to microwave drying/curing was most often higher than in samples subjected to conventional drying (4.66 vs 1.93) (Table 1). Both groups of samples tested after the 10 HDLW showed the similar growth value (eg, 2.87 and 2.67) (G) measured on the antibacterially treated sample and insufficient activity values (A) (eg, -0.56 and -0.36), which means that growth value of the control fabric was smaller than the growth value of the antibacterially treated sample, showing that there was no antibacterial activity left on cotton fabric.

**Figure 2:**
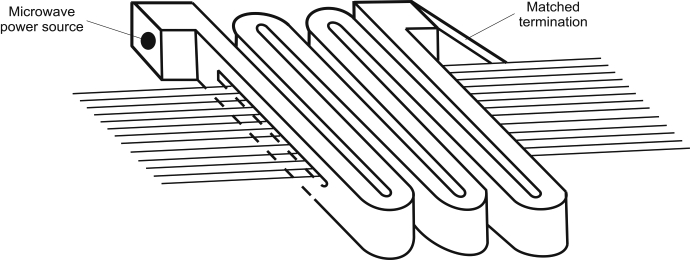
Field emission scanning electron microscope micrograms of cotton materials cured by microwaves (**A**) untreated control cotton material; (**B**) cotton material treated with 6.9% citric acid and 6.5% sodium hypophosphite monohydrate prior to home domestic laundering washing; (**C**) cotton material treated with 6.9% citric acid and 6.5% sodium hypophosphite monohydrate after 10 home domestic laundering washing cycles.

The surfaces of the cotton fibers before and after the antimicrobial treatment were morphologically observed by scanning electron microscopy (SEM). Figure 2 shows SEM micrographs of control samples (Figure 2A) and cotton material treated with CA and SHP prior to washing (Figure 2B) and subjected to 10 HDLW cycles (Figure 2C).

The surface of the untreated cotton polymer (Figure 2A) was smooth, while minor erosion stripes appeared on the surfaces treated with antimicrobial agent (Figure 2B). Furthermore, on the surface of the fibers treated with CA, a small amount of the deposited solid material was noticed, probably due to the presence of the unwashed SHP catalyst and excess CA. Long and deep stripes were found on the surface of washed sample (Figure 2C), which is attributed to the damage that occurred during the tumbling process.

**Figure 3:**
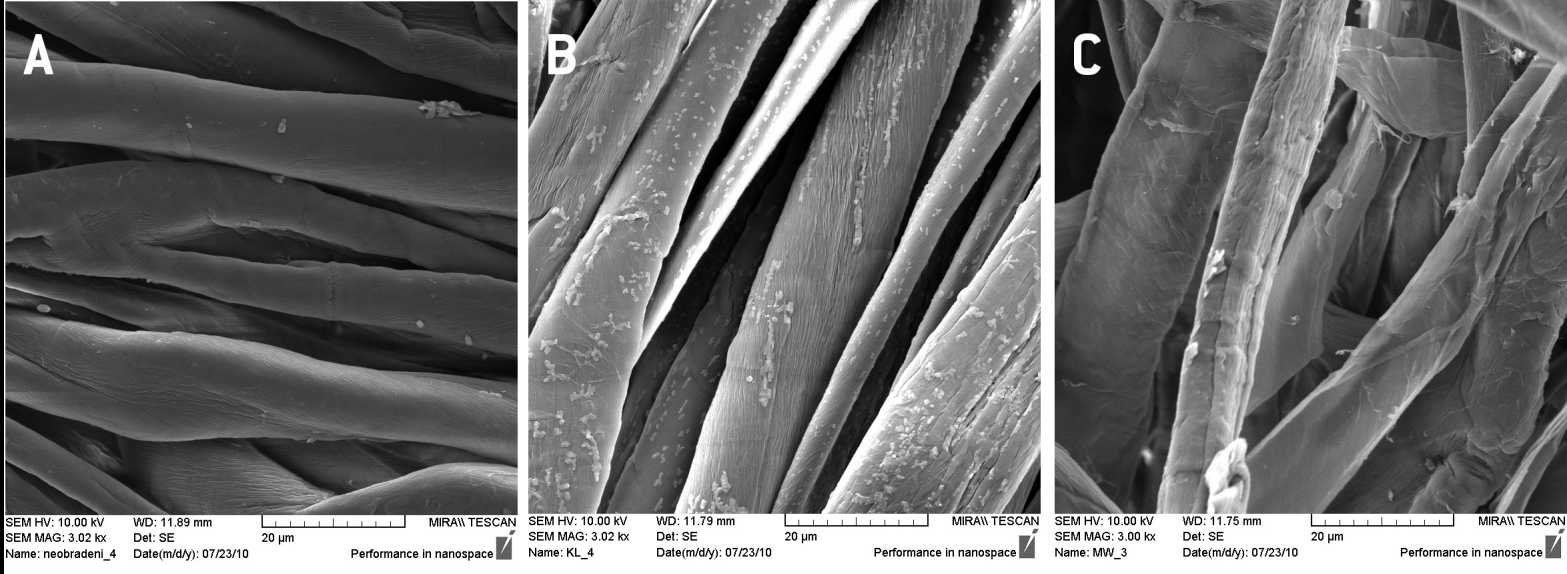
Wrinkle recovery angles (WRA), presented as sum of warp and fill (W+F) for determination of ester linkages quantity of untreated-cotton fabric and cotton fabric treated with 6.9% citric acid (CA) and 6.5% sodium hypophosphite monohydrate before home domestic laundering washing (0 HDLW), after 1 cycle of HDLW (1HDLW), and after 10 cycles of HDLW cured conventionally (closed bars) and by microwaves (open bars).

**Figure 4:**
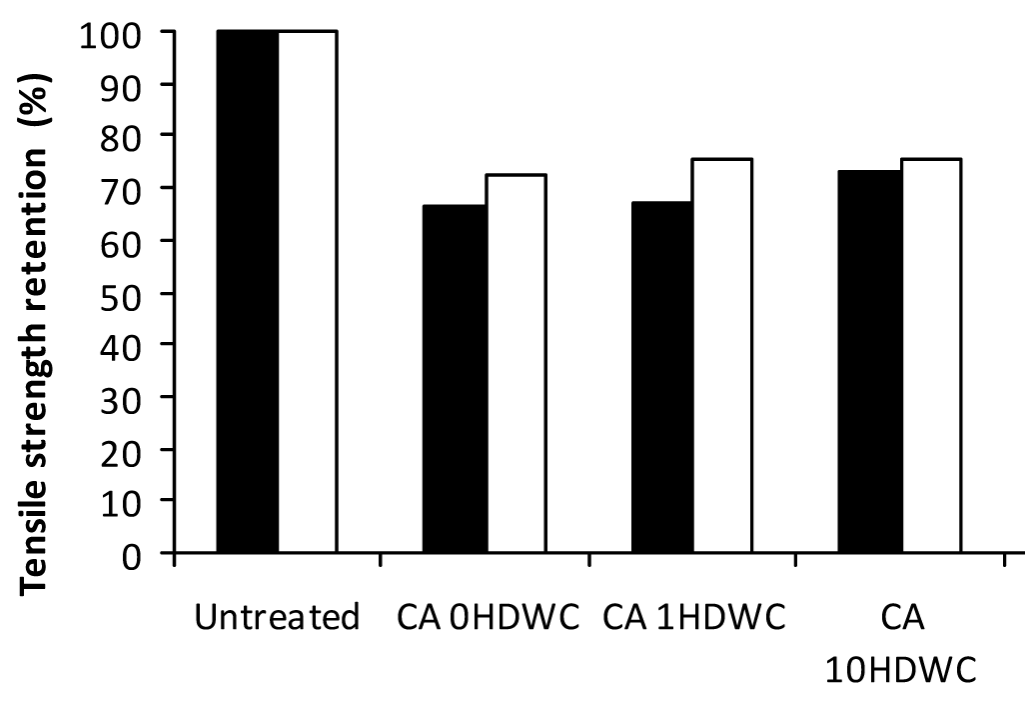
Tensile strength retention (%) of untreated-cotton fabric and cotton fabric treated with 6.9% citric acid (CA) and 6.5% sodium hypophosphite monohydrate prior to home domestic laundering washing (0HDLW), after 1 cycle of HDLW (1HDLW), and after 10 cycles of HDLW cured conventionally (closed bars) and by microwaves (open bars)

One of the indirect methods for determination of ester linkages quantity is the wrinkle recovery angle (WRA) method (Figure 3), while tensile strength method estimates whether thermal treatment caused changes in the microstructure (Figure 4).

The difference in performance between the conventional and microwave system becomes quite clear when evaluating wrinkle resistance (Figure 3). Microwave method of curing offered significant improvements in WRA (226° in comparison to 178° obtained by conventional system).

The spectroscopy analysis is widely used for qualitative and quantitative determinations of ester linkages formed between the hydroxyls of CA and cellulose. The results (Table 2) showed that Whiteness Index of the International Commission on Illumination (*Commission internationale de l'éclairage*) (W_CIE_) increased linearly with the increased number of washing cycles. After 10 washing cycles, W_CIE_ increased up to the value of 111, while the value of the untreated material was 135. Regarding the evaluation of the W_CIE_ and yellowness index (Table 2), the changes were more prominent when microwave treatment was used. Obviously, microwave treatment results in a decrease in the W_CIE_ already after the first laundering cycle. During the treatment of fabrics with CA at elevated curing temperatures, both with the conventional and microwave curing, the problem of yellowness occurred, with higher yellowness index in treated than in untreated samples (Table 2).

**Table 2:** Whiteness and yellowness index of cotton samples treated with 6.9% citric acid (CA) and 6.5% sodium hypophosphite monohydrate (SHP) after different number of washing cycles measured according to AATCC method 110-2005 (“Whiteness of textiles”) (19)*

	Conventional treatment		Microwave treatment	
	WI_CIE_	YI	WI_CIE_	YI
Untreated	135.0	-19.84	135.0	-19.84
CA + SHP:				
0 HDWC cycles	89.7	-2.21	78.2	-0.21
1 HDWC cycle	92.4	-3.24	86.3	-2.54
10 HDWC cycles	111.4	-10.54	105.1	-8.34

*Abbreviations: WI_CIE_ – Whiteness Index of the International Commission on Illumination (*Commission internationale de l'éclairage*); YI – yellowness index; HDWC – home domestic laundering washing.

## Discussion

Antibacterial treatment of cotton carbohydrate polymer with CA and its SHP catalyst proved to be a viable anti-MRSA technique and the fabrics demonstrated excellent antibacterial activity values after the exposure to the both *S. aureus* strains and *P. aeruginosa* strain.

Despite stringent control measures in endemic settings, bacterial eradication is very hard to achieve. Nosocomial infections have also been viewed as serious preventable infections, partly because they do not simply replace the infections resulting from susceptible strains but actually add to nosocomial infection rates (2). In order to improve the current protocols and initiatives in infection control, the use of antimicrobial agents applied on cotton carbohydrate polymer should be considered. When antimicrobial agents are applied to the staff uniforms, they can actually prevent the transmission of *S. aureus* and *P. aeruginosa* through clothing.

The study was performed with the aim to improve hospital hygiene measures and contribute to the prevention of bacterial spread in health care facilities by the use of antimicrobial textiles. The choice of antimicrobial finishing was influenced by demands for application of environmental and eco-friendly antimicrobial finishings, and CA is already listed as an anti-MRSA agent, but when applied with a different catalyst.

As expected, antibacterial activity was reduced after HDLW, especially after 10 cycles, but the compound was still effective. To satisfy the regulations of ISO 20743:2009 standard, it is necessary that the growth value obtained according to the above presented formula is equal to or greater than 1.0 in the plate count method.

Microwave treatment, compared with conventional treatment, for drying/curing, exhibited moderate increase in antibacterial activity, especially for *P. aeruginosa* strain. Furthermore, after the microwave treatment, the number of cross-links, measured by the WRA, was increased, as was the tensile strength retention. An additional advantage of microwave treatment is the lower energy consumption, while the effectiveness is equally good, in some cases even slightly better. The only disadvantage is a decrease in the whiteness index already after the first laundering cycle.

It is believed that the microwave energy heats the cloth or garment uniformly from inside out, which results in uniform distribution of cross-links and polymerization throughout the microstructure (19). Furthermore, uniformity of such heating prevents serious losses in strength, while still imparting the desired crease-resistant durable press properties. This offers considerable advantages compared with conventional surface heating.

The results of durable antibacterial activity confirmed that the agent was able to form covalent bonds with the cellulose hydroxyls, which was previously confirmed with the Fourier Transform Infrared analyses (20).

The problem of yellowness, which might occur during the treatment with CA at elevated curing temperatures, both during the conventional and microwave treatment, has been known from our earlier research (21). The presence of the hydroxyl group may cause a noticeable yellowing of the material during the heat cure (14). Decrease in W_CIE_ is, as expected, the most noticeable with the application of CA, but the W_CIE_ results were already increased again after the first washing cycles, which is of great esthetic importance.

The application of microwave treatment revealed equally good or slightly better effectiveness than the conventional method, while its major advantage lies is in the lower energy consumption and therefore can certainly be recommended as environmentally friendlier procedure.

CA is safe in process and use, and can be applied easily and economically on textile fabrics. It is considered to be environmentally benign and reduce the burden of nosocomial pathogens.
